# A 12-Bit High-Speed Column-Parallel Two-Step Single-Slope Analog-to-Digital Converter (ADC) for CMOS Image Sensors

**DOI:** 10.3390/s141121603

**Published:** 2014-11-17

**Authors:** Tao Lyu, Suying Yao, Kaiming Nie, Jiangtao Xu

**Affiliations:** School of Electronic Information Engineering, Tianjin University, 92 Weijin Road, Nankai District, Tianjin 300072, China; E-Mails: lvtao@tju.edu.cn (T.L.); syyao@tju.edu.cn (S.Y.); nkaiming@tju.edu.cn (K.N.)

**Keywords:** CMOS image sensor, column-parallel ADC, single-slope ADC, two-step

## Abstract

A 12-bit high-speed column-parallel two-step single-slope (SS) analog-to-digital converter (ADC) for CMOS image sensors is proposed. The proposed ADC employs a single ramp voltage and multiple reference voltages, and the conversion is divided into coarse phase and fine phase to improve the conversion rate. An error calibration scheme is proposed to correct errors caused by offsets among the reference voltages. The digital-to-analog converter (DAC) used for the ramp generator is based on the split-capacitor array with an attenuation capacitor. Analysis of the DAC's linearity performance *versus* capacitor mismatch and parasitic capacitance is presented. A prototype 1024 × 32 Time Delay Integration (TDI) CMOS image sensor with the proposed ADC architecture has been fabricated in a standard 0.18 μm CMOS process. The proposed ADC has average power consumption of 128 μW and a conventional rate 6 times higher than the conventional SS ADC. A high-quality image, captured at the line rate of 15.5 k lines/s, shows that the proposed ADC is suitable for high-speed CMOS image sensors.

## 1. Introduction

Due to their benefits of low power, low cost and flexible system integration with on-chip circuits, CMOS image sensors (CIS) have been experiencing explosive growth in recent years and have made themselves competitive to charge-coupled devices (CCD), particularly in high-speed videography. There exist three analog-to-digital converter (ADC) architectures utilized in CMOS image sensors: the single-channel ADC, the column-parallel ADC and the pixel-level ADC. The column-parallel ADC is the most widely used architecture because it provides a better tradeoff among readout speed, silicon area and power consumption. Successive approximation register (SAR) ADC [1,2], cyclic ADC [3], and single-slope (SS) ADC [4] are usually employed as the column-parallel ADC. SAR ADCs have been utilized in high-speed image sensors, but they occupy a large silicon area. Cyclic ADCs occupy less area while providing high speed, but the high-speed operation amplifier (op-amp) in each column consumes more power. SS ADCs have been most widely applied in CMOS image sensors because of their simplicity, low power consumption, high linearity, and small area. Moreover, they can ensure uniformity between columns and thus minimize column fixed-pattern noise (FPN). However, SS ADCs have a disadvantage of low conversion speed. Each *n*-bit conversion requires 2*^n^* clock periods. Although high-speed SS ADCs have been recently reported [5], they use very high clock frequency which in turn leads to high power consumption.

To solve the problem of low speed, two-step SS ADCs have been recently reported in [6–10]. In these types, the analog-to-digital (AD) conversion is carried out using two steps: a coarse conversion and a fine conversion. In [6], multiple ramp voltages are used for the fine conversion to increase the conversion rate. However, this architecture has increased area and power consumption due to the multiple ramp generators. In [7,9], it stores the analog coarse voltage on a capacitor inside the column, requiring only one ramp voltage. However, high pixel density will require the ramp generator to drive a huge overall column capacitance, substantially affecting the settling accuracy and speed and consuming high power.

This paper proposes a new two-step SS ADC using a single ramp voltage and multiple reference voltages. The proposed ADC has a significantly increased conversion rate compared with the conventional SS ADC, and costs less power and area than the multiple-ramp ADC. The remainder of this paper is organized as follows. Section 2 describes the operation principle of the proposed ADC and error calibration. Section 3 discusses the implementation details of a prototype Time Delay Integration (TDI) CMOS image sensor. Section 4 presents the experimental results, and Section 5 provides the conclusions.

## 2. Proposed Two-Step SS ADC Architecture

### 2.1. Operation Principle

**Figure 1.**
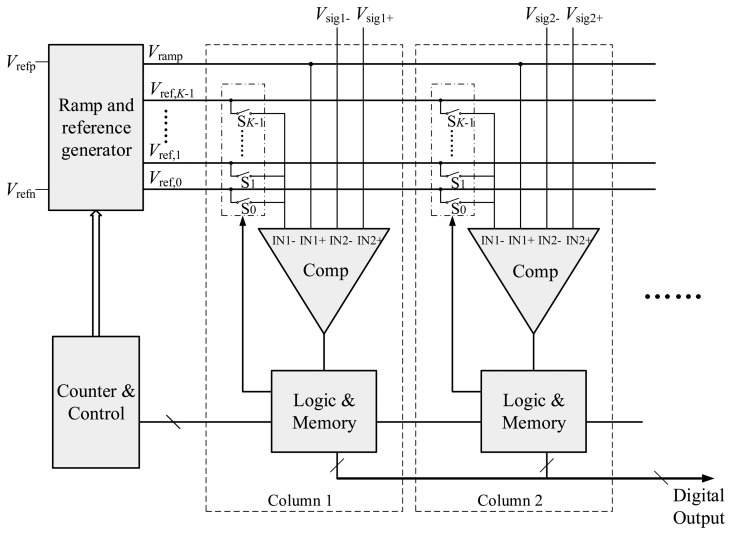
Block diagram of the proposed two-step SS ADC.

**Figure 2.**
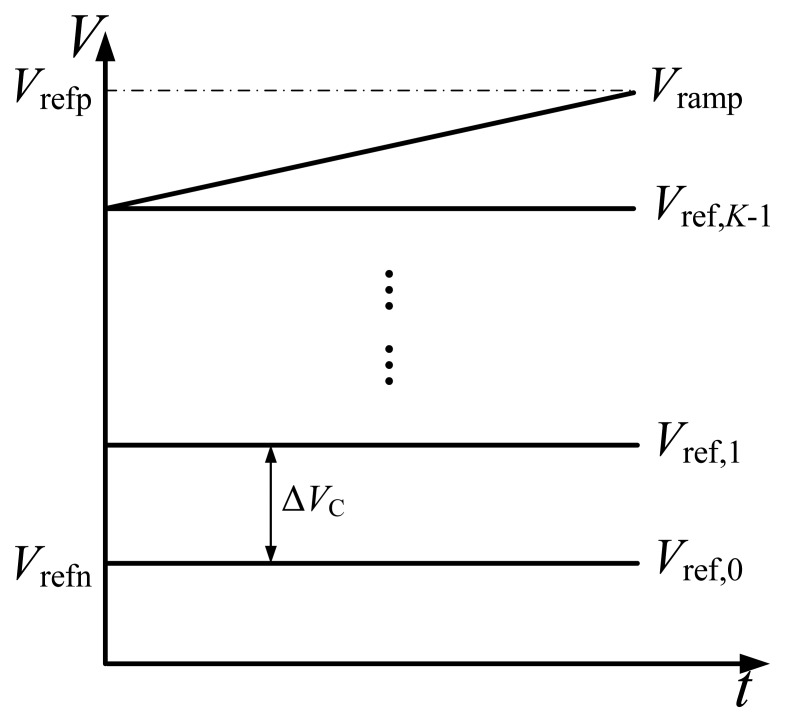
The ramp voltage and *K* reference voltages.

The basic concept of the proposed ADC is dividing the *n*-bit AD conversion into *p*-bit coarse and *q*-bit fine conversions where *n* is the sum of *p* and *q* in an ideal case. The block diagram of the proposed two-step SS ADC is shown in Figure 1. A ramp and reference generator, a counter and a control block are shared by all column circuits. The ramp and reference generator outputs one ramp voltage and *K* reference voltages to drive all the columns, where *K* = 2*^p^*. As shown in Figure 2, these *K* reference voltages divide the entire input range, *V*_ref_ = *V*_refp_ − *V*_refn_, into *K* sections evenly, each of which has a range of Δ*V*_C_ = *V_ref_*/*K*. And the ramp voltage spans 1/*K* of the input range, from *V*_ref,_*_K_*_−1_ to *V*_refp_. For simplicity, it is assumed that *V*_refp_ and *V*_refn_ are connected to *V*_ref_ and ground, respectively. Therefore, *V*_ref,_*_k_* and *V*_ramp_ can be expressed respectively in the ideal case as:

(1) $$\begin{array}{cc} V_{\text{ref},k}=k\cdot \Delta V_{\text{C}}=k\cdot 2^{q}\cdot \text{LSB}, & k=0,1,...,K-1 \end{array}$$

(2) $$\begin{array}{cc} V_{\text{ramp}}(c)=c\cdot \text{LSB}, & (K-1)\cdot 2^{q}\leq c<K\cdot 2^{q} \end{array}$$

where *c* is the counter value.

In addition, each column circuit consists of a comparator, a set of switches, logic gates and memory. The switches S_0_ ∼ S*_K−_*_1_ are used to connect one of the *K* reference voltages to the input of the comparator. Compared with a SS ADC, the proposed structure only requires a number of additional switches and some extra digital circuitry in each column.

**Figure 3.**
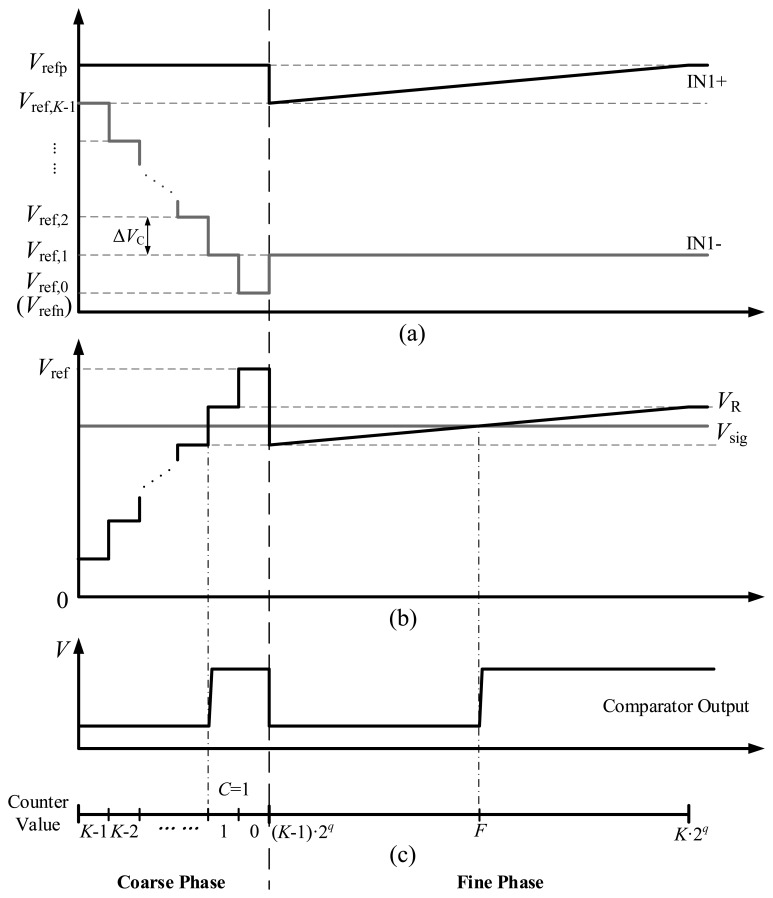
Timing diagram of the proposed two-step SS ADC. Waveforms of (**a**) the IN1+ and IN1− terminals of the comparator; (**b**) the differential voltages *V*_R_ and *V*_sig_; (**c**) the comparator output.

In Figure 3, the operation of the proposed ADC is illustrated with a timing diagram. The AD conversion is divided into coarse and fine phases. In the coarse phase, the counter value decreases from *K*−1 to 0, and the analog switches S*_K_*_-1_ ∼ S_0_ are turned on in sequence, connecting *V*_ref,_*_K_*_−1_ ∼ *V*_ref,0_ to the IN1− terminal of the comparator respectively. At the same time, the ramp voltage *V*_ramp_, which is connected to the IN1+ terminal, outputs the maximum voltage *V*_refp_. The waveforms of IN1+ and IN1− terminals are shown in Figure 3a. As a consequence, the differential voltage between IN1+ and IN1−, *V*_R_ = *V*_IN1+_ − *V*_IN1−_, is a coarse ramp signal with the step of Δ*V*_C_, as shown in Figure 3b. When *V*_R_ exceeds the input differential signal *V*_sig_ = *V*_IN2+_ − *V*_IN2−_, the comparator output changes to high level, as shown in Figure 3b,c. Then the counter value is stored in the column memory as the coarse conversion result *C*. In the case shown in Figure 3, *C* equals 1.

The fine conversion phase is then performed. The coarse conversion result *C* is fed back into the analog switches which connects the correct reference voltage to the comparator. Thus S*_C_* is closed, and the IN1− terminal is connected to *V*_ref,_*_C_*, as shown in Figure 3a. Meanwhile, *V*_ramp_ outputs a ramp signal, which spans Δ*V*_C_ from *V*_ref,_*_K_*_−1_ to *V*_refp_. As a result, *V*_R_ becomes a fine ramp signal which will intersect *V*_sig_. When *V*_R_ exceeds *V*_sig_, the comparator changes its output to low high, and the counter value is stored in the column memory as the fine conversion result *F*. Ignoring the quantization error, the comparator triggers when:

(3) $$\begin{array}{cc} V_{\text{sig}} & =V_{\text{ramp}}(F)-V_{\text{ref},C}\\ & =\left(F-C\cdot 2^{q}\right)\cdot \text{LSB} \end{array}$$

where (*K*–1) 2*^q^* ≤ *F* < *K* 2*^q^*.

Therefore, the final digital output is obtained by:

(4) $$D_{\text{out}}=F-C\cdot 2^{q}$$

The conversion time of the proposed ADC is reduced to 2*^p^* + 2*^q^* clock cycles for a n-bit AD conversion, while the conventional SS ADC requires 2*^n^* clock cycles. So the speed of the proposed structure increases greatly compared with conventional SS ADCs.

In [6], the multiple-ramp ADC employs eight ramp voltages, each of which needs a buffer with high power consumption to drive all the columns. And in this proposed ADC structure, only one ramp voltage with such a buffer is utilized. Although each reference voltage also needs a buffer, the buffer requires low unity gain bandwidth and costs low power because what it buffers is a DC voltage. Therefore, the total power cost by the buffers is much less in the proposed structure than in the multiple-ramp ADC.

### 2.2. Error Calibration

**Figure 4.**
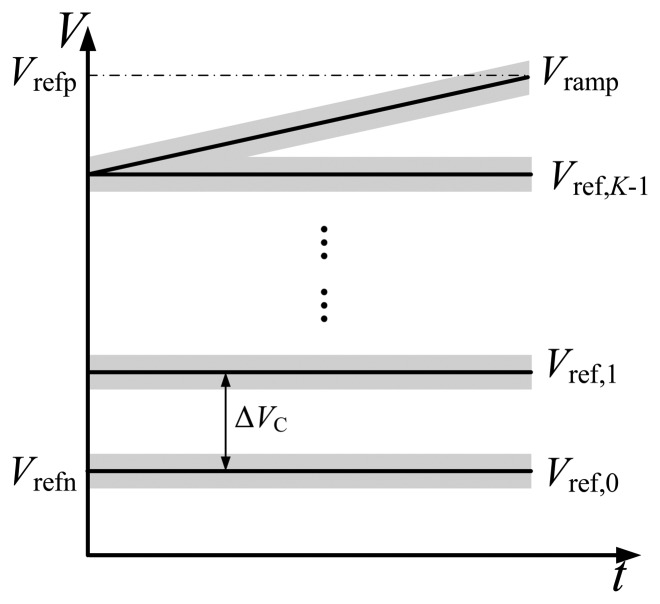
The ramp voltage and *K* reference voltages in practice.

In practice, offsets among the reference voltages and the ramp voltage will cause serious performance degradation. As shown in Figure 4, the solid lines represent the ideal reference voltages, and the shadows represent the probable range of the real reference voltages with offset. Assuming that the offset of *V*_ref,_*_k_* is *V*_offset,_*_k_* and that of the ramp voltage is *V*_offset,ramp_, Equation (3) becomes:

(5) $$\begin{array}{ll} V_{\text{sig}} & =\left[V_{\text{ramp}}(F)+V_{\text{offset},\text{ramp}}\right]-\left[V_{\text{ref},C}+V_{\text{offset},C}\right]\\ & =(F-C\cdot 2^{q})\cdot \text{LSB}+V_{\text{offset},\text{ramp}}-V_{\text{offset},C}\\ & =D_{\text{out}}\cdot \text{LSB}+V_{\text{offset},\text{ramp}}-V_{\text{offset},C} \end{array}$$

Then, the relation between the digital output *D*_out_ and input voltage *V*_sig_ can be obtained as follows

(6) $$D_{\text{out}}=\left(V_{\text{sig}}-V_{\text{offset},\text{ramp}}+V_{\text{offset},C}\right)/\text{LSB}$$

As we can see, the offsets would introduce errors into the digital output, and deteriorate the linearity of ADC seriously. Furthermore, the offsets will lead to uncertain shift of *V*_R_ during the fine phase, resulting in dead bands in the final digital output.

**Figure 5.**
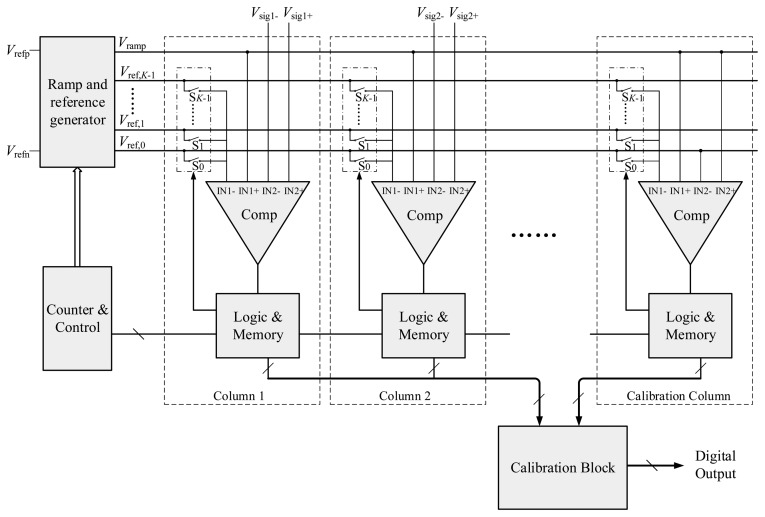
Block diagram of the proposed two-step SS ADC with auto-calibration.

This problem is corrected by the following auto-calibration algorithm. Another column circuit called the calibration column is added to the original structure. Its differential input is directly connected to *V*_ramp_ and *V*_ref,0_, as illustrated in Figure 5. A calibration block, in which the auto-calibration algorithm operates, is employed and placed on the readout bus. Besides, in order to avoid dead bands, the range of the ramp is extended, which spans from *V*_ref,_*_K_*_−1_ − Δ*V*_ex_ to *V*_refp_. This extension corresponds to the introduction of one bit redundancy in the fine phase.

In the sampling phase, the ramp generator will output a test voltage *V*_test,_*_m_*, which corresponds to the middle of each fine conversion subsection, expressed as:

(7) $$\begin{array}{cc} V_{\text{test},m}=(m+\frac{1}{2})\cdot \Delta C+V_{\text{offset},\text{ramp}}=(m\cdot 2^{q}+2^{q-1})\cdot \text{LSB}+V_{\text{offset},\text{ramp}} & m=0,1\ldots K-1 \end{array}$$

The calibration column will sample the difference between *V*_test,_*_m_* and *V*_ref,0_. The conversion process of the calibration column is the same as that of the other columns as mentioned above. In the coarse phase, the coarse result *C* will be equal to *K* – *m* − 1. The calibration column circuit will select *V*_ref,_*_C_* after the coarse phase. The subsequent fine phase will become a comparison between *V*_test,_*_m_* − *V*_ref,0_ and *V*_ramp_ − *V*_ref,_*_C_*. When the comparator triggers,

(8) $$V_{\text{test},m}-V_{\text{ref},\text{0}}=V_{\text{ramp}}-V_{\text{ref},C}$$

Similar to Equation (6), the digital output of the calibration column can be expressed as:

(9) $$\begin{array}{cc} D_{\text{out},\text{cali}} & =\left(V_{\text{test},m}-V_{\text{ref},0}-V_{\text{offset},\text{ramp}}+V_{\text{offset},C}\right)/\text{LSB}\\ & =\left[((K-C)\cdot 2^{q}-2^{q-1})\cdot \text{LSB}-V_{\text{offset},0}+V_{\text{offset},C}\right]/\text{LSB} \end{array}$$

When a general column and the calibration column have the same coarse result *C*, with the aid of Equations (6) and (9), the expression is obtained as follows:

(10) $$D_{\text{out}}-D_{\text{out},\text{cali}}+(K-C)\cdot 2^{q}-2^{q-1}=\left(V_{\text{sig}}-V_{\text{offset},\text{ramp}}+V_{\text{offset},0}\right)/\text{LSB}$$

The left side of the equal sign is regarded as the corrected digital result. The right side shows that *V*_offset,_*_C_* is removed compared to Equation (6). Although the right side still consists of the term −*V*_offset,ramp_ + *V*_offset,0_, the introduced error is constant in the entire range, and only causes offset of the overall ADC curve, which means no harm to the linearity of ADC. Besides, since these two columns have the same *C*, the final result can be calculated further as:

(11) $$\begin{array}{cc} D_{\text{final}} & =D_{\text{out}}-D_{\text{out},\text{cali}}+(K-C)\cdot 2^{q}-2^{q-1}\\ & =F-F_{\text{cali}}+(K-C)\cdot 2^{q}-2^{q-1} \end{array}$$

where *F* and *F*_cali_ are the fine ADC results of a general column and the calibration column respectively.

The auto-calibration algorithm is based on Equation (11). The calibration column samples a different *V*_test,m_ per conversion, where *m* varies from 0 to *K*−1 in sequence. The results of coarse and fine conversion are transmitted to the calibration block, where the results corresponding to each *V*_test,m_ are averaged and stored in a lookup table. Meanwhile, the results of the general columns are accessed and transmitted to the calibration block one by one. Then according to the coarse result, the calibration block will find the corresponding fine result of the calibration column from the lookup table. Finally, according to Equation (11), the auto-calibration procedure works out the final output.

**Figure 6.**
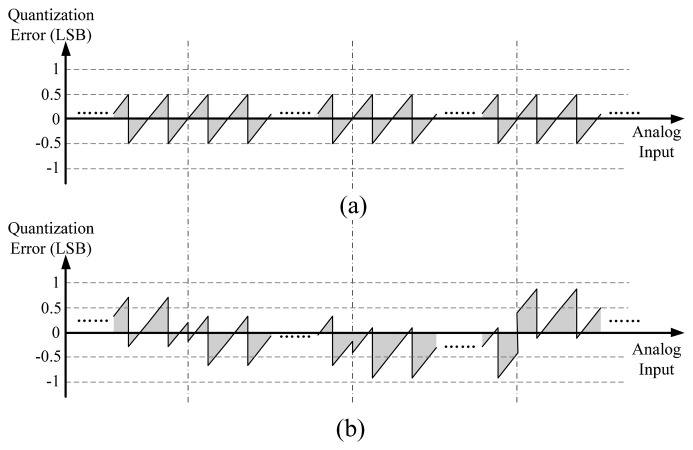
(**a**) The ideal curve of quantization error *e*_col_ *versus* input voltage; (**b**) Different parts of the curve have a uncertain shift because of *e*_cali,_*_k_*.

Although the error caused by offsets of the multiple references is corrected by the proposed scheme, the quantization error of the calibration column still affects the linearity. Taking into account the quantization error, Equation (10) becomes:

(12) $$D_{\text{final}}=\left(V_{\text{sig}}-V_{\text{offset},\text{ramp}}+V_{\text{offset},\text{0}}+e_{\text{col}}-e_{\text{cali},k}\right)/\text{LSB}$$

where *e*_col_ and *e_cali,_*_k_ are the quantization error of the general column and the calibration column respectively, which both range from −0.5 LSB to 0.5 LSB in an ideal case. Hence, the quantization error of the final output is given as *e*_out_ = *e*_col_ − *e*_cali,_*_k_*. The influence of *e_cali,_*_k_ can be illustrated in Figure 6. Figure 6a shows the ideal curve of the quantization error *e*_col_ *versus* input voltage. Because *e_cali,_*_k_ has an uncertain value for each *k*, the part of the curve corresponding to different subsection will have a uncertain and slight shift, as shown in Figure 6b. In the worst case, when *e_cali,_*_k_ is 0.5 LSB, *e_out_* may reach up to ±1 LSB, and INL will increase by 0.5 LSB. Furthermore, when *e_cali,_*_k−_*_1_* and *e_cali,_*_k_ are ±0.5 LSB and ∓0.5 LSB respectively, DNL will get worse by 1 LSB.

## 3. Implementation

### 3.1. Proposed Image Sensor Architecture

**Figure 7.**
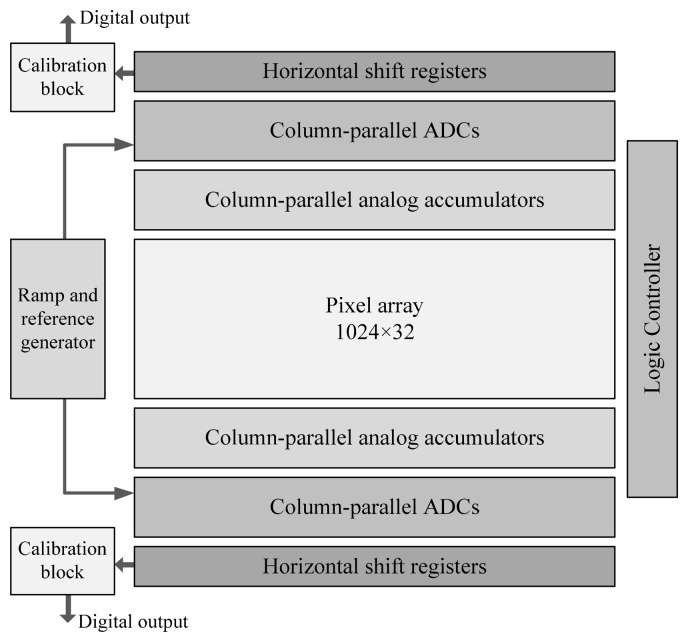
Block diagram of the proposed image sensor.

A prototype 32-stage TDI image sensor with the proposed ADC architecture has been implemented in a standard 0.18 μm CMOS process. The TDI camera is a special type of line-scan camera, which captures images through an array of pixels operating in line-scan mode [11,12]. Due to the special integration process, the camera could produce high-quality and low noise images with high scanning speed, even under low illumination conditions. In Figure 7, a block diagram of the imager is depicted. The prototype consists of a 1024 × 32 pixel array, column-parallel analog accumulators, column-parallel ADCs, a ramp and reference generator, logic controllers, horizontal shift registers and calibration blocks. Each column has an analog accumulator [11] to perform the 32-stage TDI operation. Then the accumulator's output is quantized by the proposed column-parallel ADC. The ADC resolution is 12-bit. Finally, all the conversion results will be shifted out by horizontal shift registers and transmitted to the calibration block.

As mentioned previously, the AD conversion is divided into *p*-bit coarse and *q*-bit fine conversions. The choice for *p* and *q* needs to be considered seriously. The minimum conversion time occurs when *p* = *q* = 6 for 12-bit resolution. However, such a choice for *p* and *q* implies that 64 reference voltages are required. Some practical problems make it difficult to implement such a large number of references. First, each reference voltage needs a signal line connected to all columns and a switch in each column. Therefore, too many references would occupy too much area. A second limitation stems from the fact that, offsets between multiple references may result in dead bands in the digital output. This problems can be solved by extending the range of the ramp and creating some overlap. The amount of overlap is fixed and depends on the expected magnitude of offsets. As a result, increasing the number of references will increase the proportion of the overlap to the ramp.

Based on these limitations, the 12-bit conversion is divided into 3-bit coarse and 10-bit fine conversions in the prototype, which means that eight references are used and one bit redundancy is introduced to the fine phase for calibration.

### 3.2. Ramp Generator

**Figure 8.**
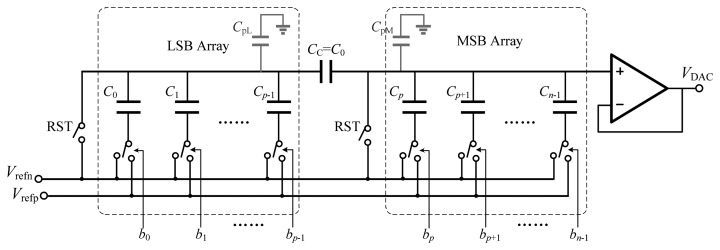
DAC based on the unary-weighted split-capacitor array.

The performance of the ramp generator determines the accuracy and linearity of the proposed ADC. The digital-to-analog converter (DAC) architecture used for the ramp generator is based on the binary-weighted split-capacitor array with an attenuation capacitor [13,14], and is illustrated in Figure 8. The capacitive-array DAC needs zero quiescent current, and capacitors match better than resistors. By employing the attenuation capacitor, the total capacitance is reduced dramatically. The *n*-bit capacitive-array DAC is split into *p* LSBs and *q* = *n* − *p* MSBs by the attenuation capacitor. Thus, the total capacitance is reduced to 2*^p^* + 2*^q^* − 1 unit capacitors compared with 2*^n^* in the conventional structure.

The value of each capacitor is given by:

(13) $$\begin{array}{l} C_{i}\;=\left\{ \begin{array}{l} \begin{array}{cc} 2^{i}\cdot C_{0} & 0\leq i\leq p-1 \end{array}\;\\ \begin{array}{cc} 2^{i-p}\cdot C_{0} & p\leq i\leq n-1 \end{array} \end{array}\right.\\ C_{\text{C}}=C_{0} \end{array}$$

where *C*_0_ is the unit capacitance. Unlike the conventional split-capacitor array in [13], the attenuation capacitor is a unit capacitor instead of fractional capacitance, which will match well with other capacitors.

**Figure 9.**
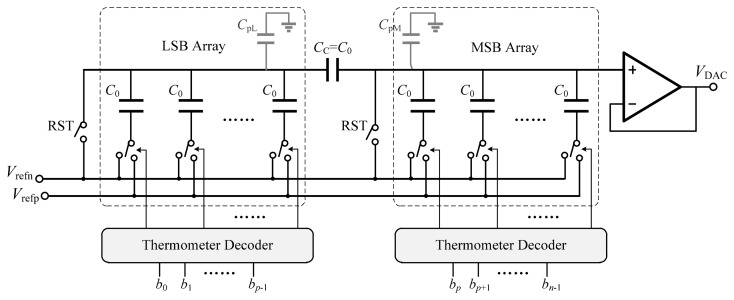
DAC based on the unary-weighted split-capacitor array.

Figure 9 shows an alternative to the structure mentioned above [14]. The unary-weighted split-capacitor array adopts the thermometer code instead of the binary code to control the switches of the capacitor array. This structure has the advantage of low differential nonlinearity (DNL) and also guarantees the monotonicity of the ramp. In addition, there is only one-bit change every time except when LSBs code changes from 111…11 to 000…00, so the jitter of the ramp voltage is relatively small.

The capacitor mismatch and the parasitic capacitance, which affect the linearity characteristics such as integral nonlinearity (INL) and DNL, are dominant factors for the medium-resolution DAC. Therefore, first a comparative analysis of the linearity due to capacitor mismatch will be presented, and the standard deviation of INL and DNL will be calculated *versus* the standard deviation of the capacitance variation. Then, the effect of parasitic capacitance on the linearity behavior will be discussed.

For the binary-weighted structure, the output voltage of the DAC is calculated from:

(14) $$V_{\text{DAC}}=\frac{V_{\text{refp}}-V_{\text{refn}}}{C_{\text{L}}+(1+\frac{C_{\text{L}}}{C_{\text{C}}})C_{\text{M}}}\left[\sum_{i=0}^{p-1}C_{i}b_{i}+(1+\frac{C_{\text{L}}}{C_{\text{C}}})\sum_{i=p}^{n-1}C_{i}b_{i}\right]+V_{\text{refn}}$$

where *C*_L_ and *C*_M_ are the total capacitance of the LSB and MSB array respectively:

(15) $$\begin{array}{ll} C_{\text{L}}\; & =\sum_{i=0}^{p-1}C_{i}+C_{\text{pL}}\\ C_{\text{M}} & =\sum_{i=p}^{n-1}C_{i}+C_{\text{pM}} \end{array}$$

where *C*_pL_ and *C*_pM_ are the parasitic capacitance in the LSB and MSB array respectively.

When ignoring the capacitor mismatch and the parasitic capacitance, Equation (14) becomes:

(16) $$V_{\text{DAC}}=\frac{V_{\text{refp}}-V_{\text{refn}}}{2^{n}-1}\cdot \sum_{i=0}^{n-1}b_{i}\cdot 2^{i}+V_{\text{refn}}$$

In order to analyze the effect of the capacitor mismatch on the linearity performance of the capacitive-array DAC, the parasitic capacitance is ignored, and each capacitor is modeled as the sum of nominal capacitance value and an error term [15,16]. Therefore, for the binary-weighted structure, each capacitor is obtained from:

(17) $$\begin{array}{l} C_{i}\;=\left\{ \begin{array}{l} \begin{array}{cc} 2^{i}\cdot C_{0}\;+\delta_{i} & 0\leq i\leq p-1 \end{array}\;\\ \begin{array}{cc} 2^{i-p}\cdot C_{0}+\delta_{i} & p\leq i\leq n-1 \end{array} \end{array}\right.\\ C_{\text{C}}=C_{0}+\delta_{0} \end{array}$$

where *δ_i_* is a random variable with a zero mean and a variance of:

(18) $$\sigma_{i}^{2}=E[\delta_{i}^{2}]=\left\{ \begin{array}{l} \begin{array}{cc} 2^{i}\cdot \sigma_{0}^{2} & 0\leq i\leq p-1 \end{array}\\ \begin{array}{cc} 2^{i-p}\cdot \sigma_{0}^{2} & p\leq i\leq n-1 \end{array} \end{array}\right.$$

where *σ*_0_ is the standard deviation of the unit capacitance.

Using the method presented in [15], the variance of the INL and DNL can be calculated from:

(19) $$\sigma_{\text{INL}}^{2}\approx (2^{2n}+2^{n+p}+2^{2n-p})\cdot {\left(\frac{\sigma_{0}}{C_{0}}\right)}^{2}\;{\text{LSB}}^{2}$$

(20) $$\sigma_{\text{DNL}}^{2}\approx 2^{n+p}\cdot {\left(\frac{\sigma_{0}}{C_{0}}\right)}^{2}\;{\text{LSB}}^{2}$$

Similarly, for the unary structure, the output can be also obtained from Equations (14) and (16). The variance of the INL and DNL can be calculated from:

(21) $$\sigma_{\text{INL}}^{2}\approx (2^{2n}+2^{n+p}+2^{2n-p})\cdot {\left(\frac{\sigma_{0}}{C_{0}}\right)}^{2}\;{\text{LSB}}^{2}$$

(22) $$\sigma_{\text{DNL}}^{2}\approx 2^{2p+1}\cdot {\left(\frac{\sigma_{0}}{C_{0}}\right)}^{2}\;{\text{LSB}}^{2}$$

In summary, from Equations (19)–(22), it can be concluded that the variance of INL in the binary structure is the same with that in the unary structure, while the variance of DNL in the unary structure is smaller by a factor of 2*^n^*^−^*^p^*^−1^ in comparison with the binary one. Therefore, the unary structure has a better linearity performance than the binary one.

In order to analyze the effect of the parasitic capacitance on the linearity characteristics, the capacitor mismatch is ignored. For the binary-weighted structure, INL can be calculated as:

(23) $$\begin{array}{ll} \text{INL} & =\frac{V_{\text{DAC},\text{real}}-V_{\text{DAC},\text{ideal}}}{\text{LSB}}\\ & =\frac{D_{\text{M}}\cdot (2^{p}-1)-D_{\text{L}}\cdot (2^{n-p}-1)}{2^{n}-1+\frac{C_{\text{pL}}}{C_{0}}(2^{n-p}-1)}\cdot \frac{C_{\text{pL}}}{C_{0}} \end{array}$$

where:

(24) $$\begin{array}{cc} D_{\text{M}}=\sum_{i=p}^{n-1}b_{i}2^{i-p}, & D_{\text{L}}=\sum_{i=0}^{p-1}b_{i}2^{i} \end{array}$$

It can be seen that the maximum positive and negative INL occur in the case of *D*_M_ = 2*^n^*^−^*^p^* − 1, *D*_L_ = 0 or *D*_M_ = 0, *D*_L_ = 2*^p^* − 1, respectively. And they have the same absolute value as:

(25) $$\left|\text{INL}\right|=\frac{\left(2^{n-p}-1)(2^{p}-1\right)}{2^{n}-1+\frac{C_{\text{pL}}}{C_{0}}\cdot (2^{n-p}-1)}\cdot \frac{C_{\text{pL}}}{C_{0}}$$

*C*_pL_ can be given as:

(26) $$C_{\text{pL}}=\alpha \cdot (\sum_{i=0}^{p-1}C_{i}+C_{\text{C}})=2^{p}\cdot \alpha \cdot C_{0}$$

where *α* is the ratio of the parasitic capacitance to a capacitor, which is mainly dependent on the process. Therefore, Equation (25) becomes:

(27) $$\left|\text{INL}\right|\approx \frac{\left(2^{n}-2^{p})\cdot (2^{p}-1\right)}{2^{n}}\cdot \alpha$$

On the other hand, the maximum DNL is expected to occur when the binary code changes from [*D*_M_,11…1] to [*D*_M_ + 1,00…0], which can be expressed as:

(28) $$\begin{array}{ll} \text{DNL} & =\frac{V_{\text{DAC}}(D_{\text{M}}+1,\;00\ldots 0)-V_{\text{DAC}}(D_{\text{M}},\;11\ldots 1)}{\text{LSB}}-1\text{LSB}\\ & =\frac{C_{\text{pL}}}{C_{0}}\;=2^{p}\cdot \alpha \end{array}$$

For the unary structure, the effect of the parasitic capacitance on the linearity characteristics is identical to that in the binary-weighted array, indicated by Equations (27) and (28).

**Figure 10.**
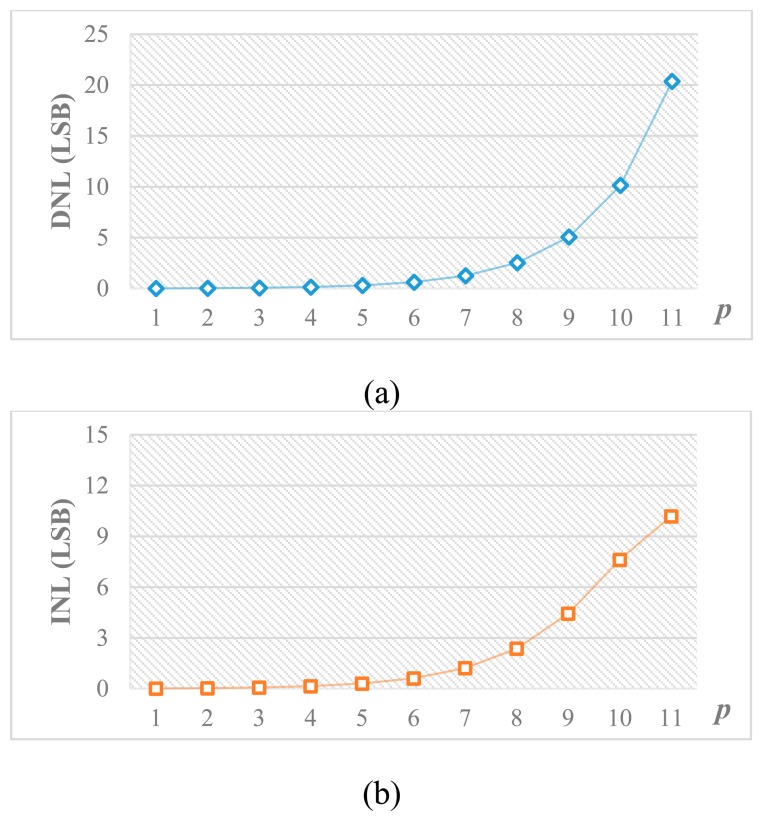
Behavior simulation results of (**a**) DNL and (**b**) INL *vs. p*.

**Figure 11.**
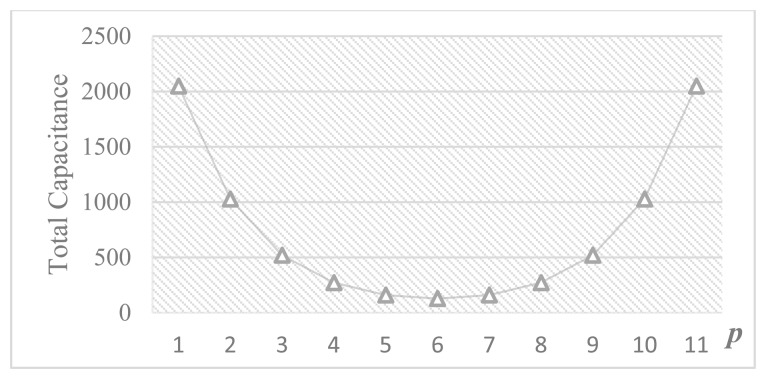
The total number of unit capacitors *vs. p*.

According to Equations (14), (25) and (28), it should be noted that *C*_pL_ degrades the linearity performance whereas *C*_pM_ only causes a gain error without affecting the linearity. Therefore, the top plate of the attenuation capacitor, which has less parasitic capacitance, is connected to the LSB array to reduce *C*_pL_. Besides, from Equations (27) and (28), it can be concluded that the linearity is dependent on *p*. Figure 10 shows the behavioral simulation results of DNL and INL caused by the parasitic capacitance *versus p* for a 12-bit DAC when *α* = 1%. By reducing *p*, the nonlinearity effect can be alleviated at the cost of larger capacitance. Figure 11 shows the total number of unit capacitors *versus p*. Thus, the distribution of bits in MSB and LSB arrays should take into account the tradeoff between linearity tolerance and total capacitance.

Based on the above discussion, a 12-bit unary-weighted split-capacitor array with an attenuation capacitor is employed in this implement, which is split into 6-bit LSB and 6-bit MSB arrays. Thus, only 127 unit capacitors are required.

**Figure 12.**
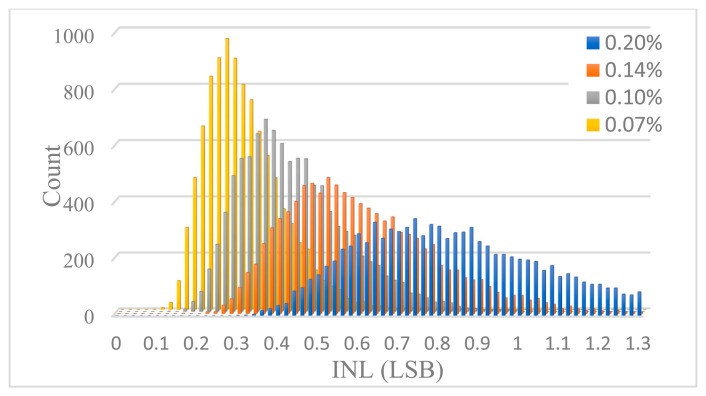
INL Histogram of 10,000 Monte Carlo simulations for some different *σ*_0_/*C*_0_.

**Figure 13.**
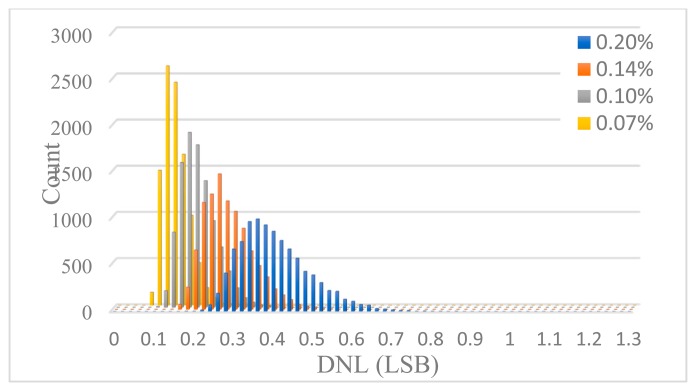
DNL Histogram of 10,000 Monte Carlo simulations for some different *σ*_0_/*C*_0_.

**Figure 14.**
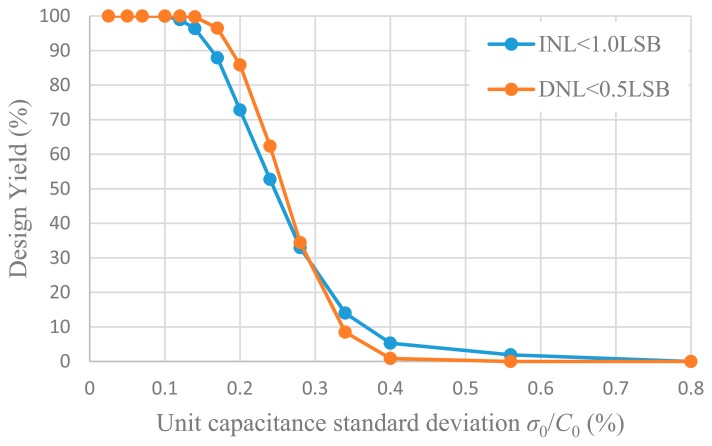
Design yield as a function of the unit capacitance standard deviation *σ*_0_/*C*_0_.

The minimum value of the unit capacitor *C*_0_ is determined by the matching requirement of capacitors, which can be estimated through Monte Carlo simulation. Each unit capacitor is taken to be independent identically distributed Gaussian random variable with a standard deviation of *σ*_0_/*C*_0_ which is regarded as the capacitance mismatch. For different *σ*_0_/*C*_0_, 10,000 Monte Carlo simulations were performed to figure out DNL and INL. Figure 12 shows the INL histogram of 10,000 simulations for some different *σ*_0_/*C*_0_, and Figure 13 shows the DNL histogram. The design yield is defined as the probability of the DAC complying with INL < 1 LSB or DNL < 0.5 LSB. The design yield as a function of the unit capacitance standard deviation is shown in Figure 14. It can be seen that with a standard deviation of 0.1%, a 99% yield for INL < 1 LSB and 100% yield for DNL < 0.5 LSB can be guaranteed. Therefore, the unit capacitor of 2 pF is selected corresponding to the standard deviation of 0.1%.

### 3.3. Reference Generator

**Figure 15.**
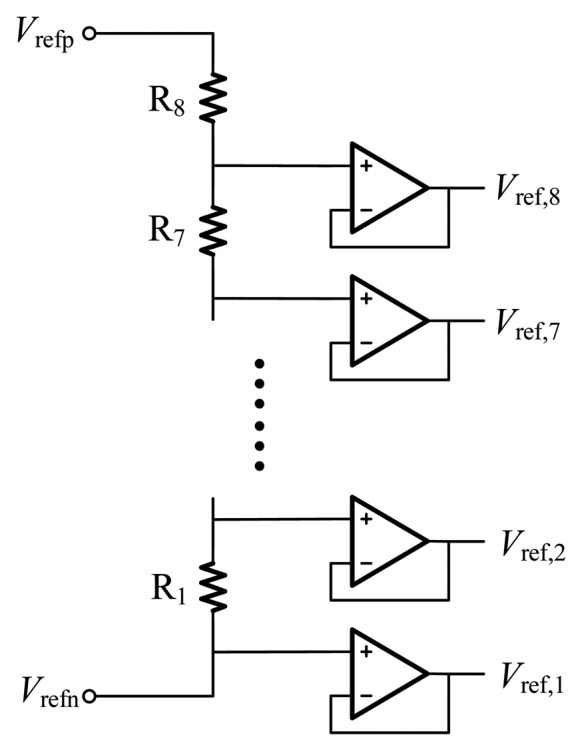
The reference generator based on a resistor string.

The reference generator is implemented based on a resistor string, as shown in Figure 15. A string of eight equal resistors, connected between two reference voltages, acts as voltage dividers to generate eight reference voltages. Then the reference voltages are buffered by folded cascade op-amps to drive all the column circuits. Though the mismatch of resistors and the offset of these buffers give rise to offsets between the reference voltages, the offsets is corrected by the calibration algorithm mentioned above. Therefore, the matching of resistors doesn't need to be considered seriously.

Though the reference generator will take more chip area and power consumption compared with SS ADC, the reference generator is shared by all columns. As a result, the average area and power consumption of each column is quite low. Besides, the total power cost by the buffers is much less in the proposed structure than in the multiple-ramp ADC. In [6], the multiple-ramp ADC employs eight ramp voltages, each of which needs a buffer with high power consumption to drive all the columns. In this structure, the buffer of each reference voltage requires low unity gain bandwidth and costs low power because what it buffers is a DC voltage.

### 3.4. Column Circuits

**Figure 16.**
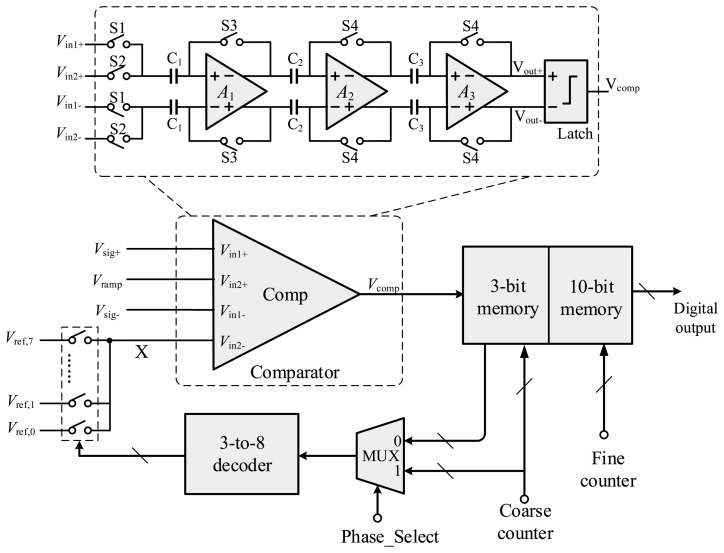
Simplified block diagram of the column circuitry.

In Figure 16, a simplified block diagram of the column-level circuitry is depicted. Each column consists of a comparator, a set of switches, logic gates and memory. Eight reference voltages *V*_ref,0_ ∼ *V*_ref,7_ are connected to the comparator *via* a 3-to-8 decoder. The output of the comparator is connected to the memory. Compared with a SS ADC, the column circuit of the proposed ADC only requires a number of additional switches and some extra digital circuitry.

In the coarse phase, the Phase Select signal is set to high level, and the multiplexer (MUX) connects the coarse counter to the 3-to-8 decoder. The analog switches are turned on in sequence corresponding to the counter value, generating a decreasing coarse ramp signal at node X. The results of the coarse conversion are stored in the 3-bit column memory. Then, the fine conversion is performed. The Phase Select signal changes to low level, and the coarse result in 3-bit memory is fed back into the analog switches *via* the MUX and the decoder. As a result, each comparator is connected to the correct reference voltage. Then when the comparator triggers, the results are stored in the 10-bit column memory, where an extra bit is used for calibration.

**Figure 17.**
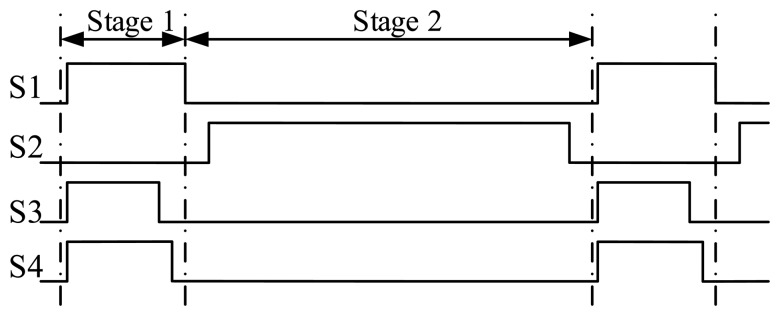
Timing diagram of the proposed comparator.

The column comparator uses three cascaded low-gain amplifiers as the preamplifier and a latch at the output, applying a dynamic offset cancellation technique [14,17]. The timing diagram of the comparator is shown in Figure 17. During Stage 1, S1, S3 and S4 are closed. The comparator is auto-zeroed, and in the meantime the input voltage *V*_in1+_ − *V*_in1−_ is sampled on C1. During Stage 2, S2 is closed to perform the comparison. Allowing for offsets of every stage, the transfer function of the three-stage preamplifier can be given as:

(29) $$V_{\text{out}+}-V_{\text{out}-}=A_{1}A_{2}A_{3}\left[\left(V_{\text{in1}+}-V_{\text{in1}-}\right)-\left(V_{\text{in2}+}-V_{\text{in2}-}\right)\right]+\frac{A_{3}}{A_{3}+1}V_{\text{OS3}}$$

where *A*_1_, *A*_2_ and *A*_3_ are gains of the three cascaded amplifiers respectively, and *V*_OS3_ is the input-offset voltage of the third amplifier. Thus, the equivalent input-offset voltage of the preamplifier is given by:

(30) $$V_{OS}=\frac{V_{OS3}}{A_{1}A_{2}(A_{3}+1)}$$

As a result, the input offset of the comparator is quite small.

**Figure 18.**
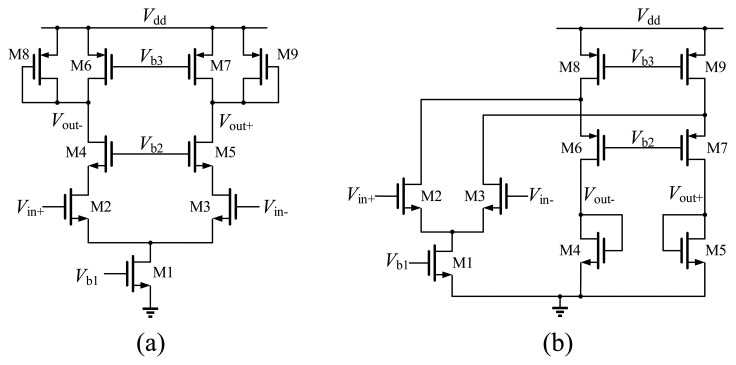
(**a**) Schematic of each amplifier in the conventional structure; (**b**) Schematic of the improved amplifier for the first stage.

In general, the three stages of the preamplifier are designed with the same schematic [14], as shown in Figure 18a. Differential input transistors M2 and M3 are loaded with diode-connected transistors M8 and M9, which can avoid common-mode feedback (CMFB). Two current sources M6 and M7 are used to enhance the gain. Transistors M4 and M5 are used to isolate the input from rapid changes in the latch output and reduce the Miller capacitance at the input. However, for the first stage, the common-mode input range is not large enough. If the input voltage exceeds the range, its gain will be degraded to even less than 1. For this reason, the first stage is designed as a folded amplifier, which has a larger common-mode input range, as depicted in Figure 18b.

Through simulation, the gain of the preamplifier is 58 dB, and the input-offset voltage is only 128 μV which is less than 0.5 LSB. The simulation results of the comparator are listed in Table 1.

## 4. Experimental Results

**Figure 19.**
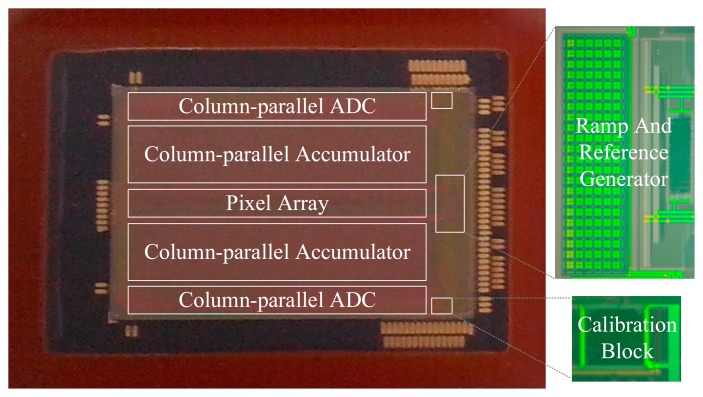
Photograph of the prototype image sensor and the chip partial microphotographs.

The prototype 32-stage TDI CMOS image sensor with the proposed two-step SS ADC architecture has been fabricated in a standard 0.18 μm one-poly four-metal 1.8 V/3.3 V CMOS process. A photograph of the image sensor and the chip partial microphotographs are shown in Figure 19. The chip size is 18.5 mm × 11.9 mm. The pixel array has 1024 × 32 4T pixels with a size of 15 μm × 15 μm and a fill-factor of 67%. The 4T pixels can achieve a higher signal-to-noise ratio (SNR) and sensitivity with a large pixel size and fill-factor under low illumination condition.

The 12-bit column-parallel ADC is divided into 3-bit coarse and 10-bit fine conversion. Each column has a layout pitch of 30 μm. Simulations of the ADC are carried out. The signal to noise and distortion ratio (SNDR) and effective number of bits (ENOB) with the proposed calibration are 72.02 dB and 11.67-bit, respectively. The ADC clock frequency is 20 MHz, and the conversion time is 36 μs. The conversion rate of the proposed ADC is improved by a factor of 6 compared with the conventional SS ADC. The average power consumption of a single column ADC is 128 μW. The power consumption of a ramp's buffer and a reference's buffer is 9.24 mW and 1.72 mW respectively. Therefore, for the multiple-ramp ADC with eight ramps, the total power consumption of all ramp's buffers is 73.92 mW, while it is significantly reduced to 23 mW for the proposed structure.

**Figure 20.**
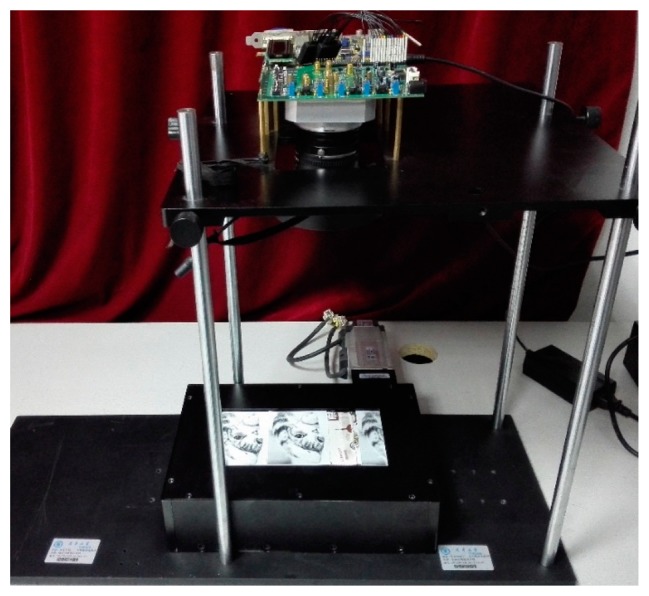
The prototype test platform.

In order to verify the TDI CIS, a prototype test platform is used, as shown in Figure 20. It is comprised of a conveyor belt, on which original photos are placed. The conveyor belt runs at a high speed when capturing images. The image data is acquired by using a logic analyzer.

**Figure 21.**
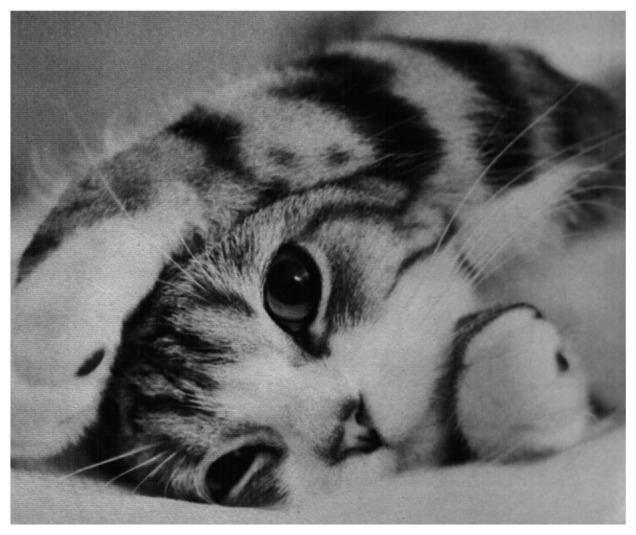
Sample image at the line rate of 15.5 k lines/s.

Figure 21 shows a sample image at the line rate of 15.5 k lines/s. However, the resolution of normal monitors is always 8-bit, so it's difficult to test the 12-bit resolution of the CIS with a monitor. In order to overcome the problems, measurements were carried out under different illumination conditions.

**Figure 22.**
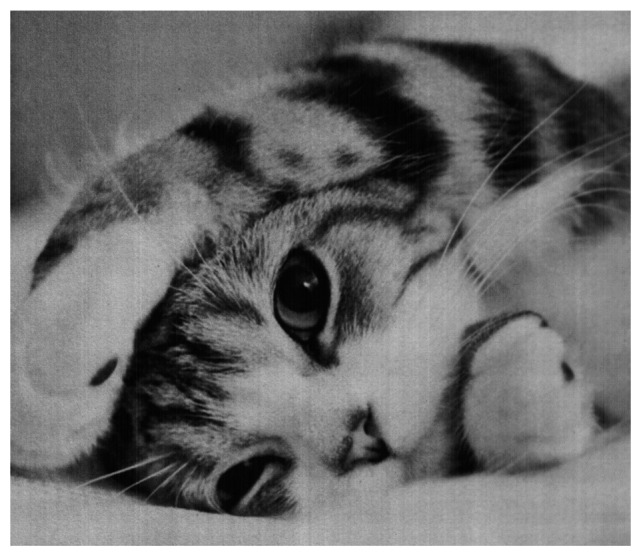
Sample image with 10-bit resolution.

**Figure 23.**
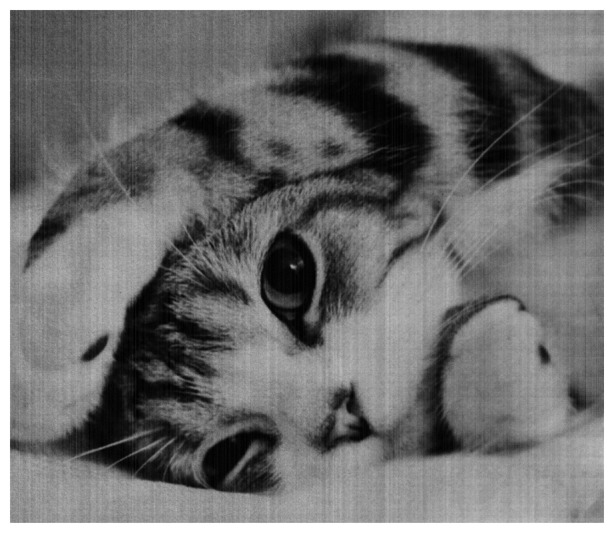
Sample image with 12-bit resolution.

When measuring under a relatively low illumination condition, all the digital outputs ranged from 0 to 2^10^. Assuming that the format of 12-bit output is [*b*_11_*b*_10_…*b*_0_], then the 8 bits, [*b*_9_*b*_8_…*b*_2_], were chose to obtain Figure 22, meaning the testing resolution is 10-bit. Under a very low illumination, the digital outputs ranged below 2^8^. Then [*b*_7_*b*_6_…*b*_0_] were chose to obtain Figure 23, meaning the testing resolution is 12-bit. In summary, high-quality images with different resolutions have been obtained with the CIS, indicating that the proposed ADC for the CIS is verified.

**Figure 24.**
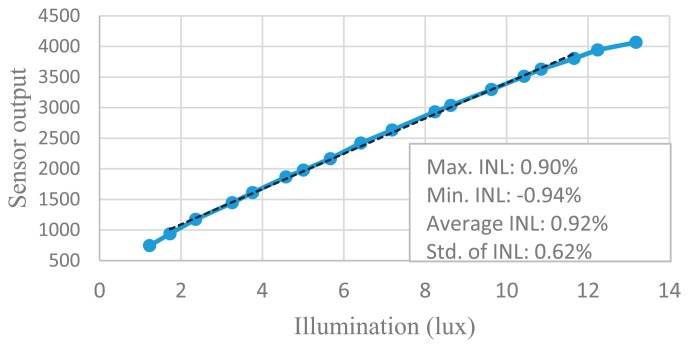
CIS output *versus* illumination and measured INL for an output range between 20% and 90% of saturation.

**Figure 25.**
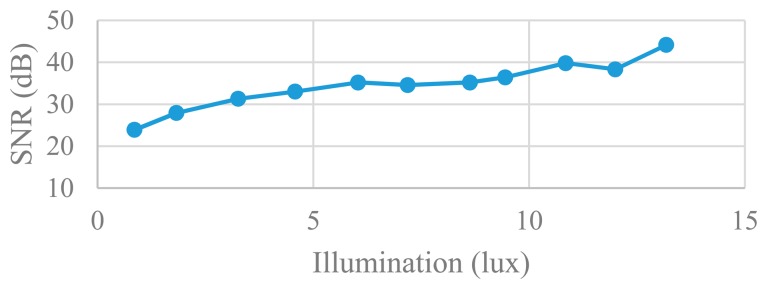
Measured SNR *versus* illumination.

The non-linearity of the CIS is measured according to the method in [18]. Figure 24 shows the CIS output *versus* illumination and measured INL for an output range between 20% and 90% of saturation. The non-linearity comes from the pixel, the analog accumulator and the column-parallel ADC. Figure 25 shows the measured SNR of the sensor *versus* illumination. In addition, the measured ADC column FPN is 0.15%, which is close to the conventional SS ADC. The performance of the proposed imager is listed in Table 2, and the comparison with other types of column-parallel ADC is listed in Table 3.

## 5. Conclusions

A 12-bit high-speed column-parallel two-step SS ADC for CMOS image sensors is proposed. The proposed ADC employs a single ramp voltage and multiple reference voltages, and the conversion is divided into coarse phase and fine phase to improve the conversion rate. An error calibration scheme is proposed to correct error caused by offsets among the reference voltages. A prototype 1024 × 32 TDI CMOS image sensor with the proposed ADC architecture has been fabricated in a standard 0.18 μm CMOS process. The proposed ADC has average power consumption of 128 μW and conversion time of 36 μs. The proposed ADC is much faster than the conventional SS ADC, and costs less power and area compared with the multiple-ramp ADC. Simulation results indicate that the SNDR and ENOB with the proposed calibration are 72.02 dB and 11.67-bit, respectively. The measured FPN is 0.15%. A high-quality image, captured at the line rate of 15.5 k lines/s, shows that the proposed ADC is suitable for high-speed CISs.

## Figures and Tables

**Table 1.** Simulation results of the comparator.

**Parameter**	**Value**
Supply voltage / V	3.3
Power / μW	90
Delay time / ns	21
Resolution / mV	0.2
Input offset / mV	0.128

**Table 2.** Performance Summary.

**Item**	**Description**
Technology	0.18-μm 1P4M CMOS
Power supply	1.8 V(Digital)/3.3 V(Analog)
Chip size	18.5 mm × 11.9 mm
Array size	1024 × 32
Pixel size	15 μm × 15 μm
Maximum line rate	15.5 k lines/s
ADC resolution	12 bit
ADC clock	20 MHz
ADC column FPN	0.15%
Column ADC pitch	30 μm
ADC input range	1.6 V
ADC conversion time	36 μs
Column ADC power	128 μW

**Table 3.** Comparison with other types of the column-parallel ADC.

**Reference**	**[14]**	**[4]**	**[6]**	**[7]**	**This Work**
Technology	0.18-μm	0.18-μm	0.25-μm	0.35-μm	0.18-μm
Pixel array	N/A	160 × 140	400 × 330	320 × 240	1024 × 32
Pixel pitch	N/A	5.6 μm	7.4 μm	5.6 μm	15 μm
Chip size	N/A	2 × 1.5 mm^2^	5 × 5 mm^2^	3.6 × 3.6 mm^2^	18.5 × 11.9 mm^2^
Architecture	Single Slope	Single Slope	Multiple-ramp SS	Two-step SS	Two-step SS
Resolution	10-bit	8-bit	10-bit	10-bit	12-bit
Conversion time	31 μs	120 μs	16 μs	4 μs	36 μs
Column power	137 μW	46 μW	95 μW	150 μW	128 μW
